# The Poincaré Half-Plane for Informationally-Complete POVMs

**DOI:** 10.3390/e20010016

**Published:** 2017-12-31

**Authors:** Michel Planat

**Affiliations:** Institut FEMTO-ST CNRS UMR 6174, Université de Bourgogne/Franche-Comté, 15 B Avenue des Montboucons, F-25044 Besançon, France; michel.planat@femto-st.fr

**Keywords:** informationally-complete POVMs, modular group, quantum computing, 03.67.-a, 03.65.Wj, 02.20.-a, 03.65.Fd, 03.65.Aa, 02.10.Ox, 03.65.Ud, 03.67.Lx, 11F06, 20H05, 81P50, 81P68, 81P13, 81P45, 20B05

## Abstract

It has been shown in previous papers that classes of (minimal asymmetric) informationally-complete positive operator valued measures (IC-POVMs) in dimension *d* can be built using the multiparticle Pauli group acting on appropriate fiducial states. The latter states may also be derived starting from the Poincaré upper half-plane model H. To do this, one translates the congruence (or non-congruence) subgroups of index *d* of the modular group into groups of permutation gates, some of the eigenstates of which are the sought fiducials. The structure of some IC-POVMs is found to be intimately related to the Kochen–Specker theorem.

## 1. Introduction

*Nulle parole ne trouve une branche où se poser (No words find a branch where to land [1])*.

Out of nothing I have created a strange new universe wrote Janos Bolyai to his father in 1823. Additionally, W. K. Clifford wrote, What Vesalius was to Galen, what Copernicus was to Ptolemy, Lobachevsky was to Euclid [2]. Half a century later, Felix Klein published the Erlangen program making explicit the relationship of all geometries to projective geometry via their own groups of symmetries. To illustrate the rise of non-Euclidean ideas in this epoch, let us quote a few words of M. Pasch to F. Klein in 1891 [3]:

*I am also late in thanking you for sending your essay on non-Euclidean geometry from last year… I also agree with most of what you say in the last two pages of your essay. The content of the axioms comes from observations (intuition as an internal activity is based on remembering what has been observed); the concepts used in the axioms, however, are inexact, and thus so are the axioms themselves. These latter can, however, only be used purely logically if they are presented as being exact. By further working on the axioms and seeking geometric propositions, we commonly make use of figures, either by drawing them or by ‘imagining’ them… The consideration must be possible even without the figures, in other words: that which is derived from the figures must already be contained in the axioms, for otherwise the axioms are not complete*.

Pasch’s axiom (of plane geometry) was used by Hilbert to complete Euclid’s axioms. It is related to Pasch’s configuration of points and lines (in projective geometry). Incidence geometry, born at the time of Pappus of Alexandria, developed with Desargues (1591–1661), Jacob Steiner (1796–1863), Thomas Kirkman (1806–1895), Gino Fano (1871–1952), David Hilbert (1862–1943) and, more recently, with Jacques Tits (1930–) and Francis Buekenhout (1937–).

What kind of relation does quantum mechanics maintain with projective geometry? The superposition of states in Hilbert space H is linear but, due to the probability interpretation of the wave function, a state is not a single vector, but a ray, i.e., a one-dimensional subspace of H. As a result, the space of rays is not a linear, but a projective space. It is known from Wigner’s theorem (1931) that the realization of symmetries for pure states of a quantum mechanical system is (up to a scalar) a unitary or anti-unitary transformation [4,5]; see also [6,7,8] for the relation to positive operator valued measures (POVMs). It is worthwhile to point out that many of the basic configurations of incidence geometry are associated to the commutation relations between Hermitian operators in the (generalized) Pauli group [9]. Some projective configurations also occur in the structure of informationally-complete positive operator valued measures (IC-POVMs) [10,11].

Section 2 is a prolegomenon to the modular group Γ, its finite index subgroups $\Gamma_{s}$ and the connection to permutation gates considered in previous papers [10,11]. Then, one deals with the concept of IC-POVMs, the relation to the Pauli group and the occurrence of the Kochen–Specker theorem. The main goal of the paper, worked out in Section 3, is the derivation of “modular” ICs that follow from the structure of Γ. In small dimensions d<10, triple products of projectors encapsulate recognizable finite geometries (already described in [11]), some of them related to the Kochen–Specker theorem. For 10≤d≤27, many ICs may be constructed thanks to appropriate subgroups $\Gamma_{s}$. A summary of the results is in Table 1.

## 2. Prolegomenon about Γ and Its Relation to IC-POVMs

### 2.1. The Modular Group Γ

The facts of science and, à fortiori, its laws are the artificial work of the scientist; science therefore can teach us nothing of the truth; it can only serve us as rule of action [12].

A standard non-Euclidean geometry consists of the Poincaré hyperbolic plane H={x,y∈R|y>0} whose symmetry group is the projective (special) linear group L=PSL(2,R) of real Möbius transformations of H. A discrete subgroup of *L* is called a Fuchsian group with the modular group Γ=PSL(2,Z) as the most celebrated example. Important mathematical objects are the moduli space of elliptic curves, which is the quotient space H/Γ, and modular forms that map pairs of points of H up to a weight factor and are also related to elliptic curves (via the 1995 modularity theorem) [13].

The modular group Γ acts discontinuously on the extended upper half-plane $H^{*}=H\cup Q\cup \infty$, i.e., for each $z\in H^{*}$, there exists a neighborhood of *z* with no other element in the orbit of *z*. Thus, Γ tessellates $H^{*}$ with infinitely many copies of the fundamental domain F={z∈Hwith|z|>1,$ℜ\left(z\right)<\frac{1}{2}$}. The modular group Γ is generated by two transformations $S_{\Gamma }:z\to -\frac{1}{z}$ and $T_{\Gamma }:z\to z+1$. It can also be represented as the two-generator free group $G=\langle e,v|e^{2}=v^{3}=1\rangle$ using the variable change $e=S_{\Gamma }$ and $v=S_{\Gamma }T_{\Gamma }$.

Some finite index subgroups of Γ, called congruence subgroups, are obtained by fixing congruence relations on the entries of elements of Γ. The principal congruence subgroup of level *N* of Γ is the normal subgroup $\Gamma \left(N\right)=\left(\begin{array}{cc} a & b\\ c & d \end{array}\right)|a,d=\pm 1\;\mathrm{mod}\;N\;\mathrm{and}\;b,c=0\;\mathrm{mod}\;N$ whose index is $n^{3}\Pi_{p|N}\left(1-\frac{1}{p^{2}}\right)$, *p* a prime number. Another important subgroup of Γ is the congruence subgroup $\Gamma_{0}\left(N\right)$ of level *N* defined as the subgroup of upper triangular matrices with entries defined modulo *N*. The index of $\Gamma_{0}\left(N\right)$ is the Dedekind psi function ψ(N). More generally, a congruence subgroup $\Gamma_{c}$ contains Γ(N) for some *N*, and the level of $\Gamma_{c}$ is the smallest positive *N* such that $\Gamma \left(N\right)⊇\Gamma_{c}$.

Important geometric invariants of a finite index subgroup $\Gamma_{s}$ of Γ (either congruence or not) are the genus, the structure of elliptic points and that of cusps (parabolic points) [17]. These concepts are defined below. A fundamental domain $F_{s}$ of $\Gamma_{s}$ in the upper half-plane H is such that for any z∈H, there is a unique $\gamma \in \Gamma_{s}$ such that $\gamma \left(z\right)\in F_{s}$. The subgroup $\Gamma_{s}$ acts discontinuously on the extended upper half-plane $H^{*}$ and tessellates it with infinitely many copies of $F_{s}$. Fixed points in H are elliptic points. An elliptic point of $\Gamma_{s}$ is a transformation $\gamma \in \Gamma_{s}$ such that γ(z)=z and γ≠±I, where *I* is the identity matrix. Elliptic points of $\Gamma_{s}$ satisfy |tr(γ)|<2, and their order can only be two or three. Their numbers are denoted $\nu_{2}$ and $\nu_{3}$, respectively. A cusp of $\Gamma_{s}$ is a fixed point of the extended upper-half plane $H^{*}$ such that |tr(γ)|=2. It can be shown that the action of $\Gamma_{s}$ partitions Q∪∞ into equivalence classes where $q_{1}∼q_{2}$ if $q_{1}=\gamma q_{2}$ for some $\gamma \in \Gamma_{s}$. These equivalence classes correspond to the cusps of $\Gamma_{s}$, and the widths of the cusps are the ratios between the orders of ${\mathrm{Stab}}_{\Gamma (q)}$ and ${\mathrm{Stab}}_{\Gamma_{s}\left(q\right)}$. The level of {q} is the least common multiple of the cusp widths of Γ. The structure of cusps is denoted $\left[\cdots c_{i}^{w_{i}}\cdots \right]$ with $c_{i}$ the number of cusps of width $W_{i}$. In Table 1, the signature of a non-congruence subgroup $\Gamma_{s}$ is represented as NC $\left(g,N,\nu_{2},\nu_{3},\left[\cdots c_{i}^{w_{i}}\cdots \right]\right)$. If the subgroup of Γ is a congruence subgroup $\Gamma_{c}$, the geometric invariants are available in [18]. To conclude this subsection, a Farey symbol for $\Gamma_{s}$ is a certain finite sequence of rational numbers (they are fractions representing vertices of a fundamental domain of $\Gamma_{s}$) together with pairing information for the edges between the vertices [17]. This concept is needed to construct the fundamental domain of a finite index subgroup of Γ.

Using methods developed in [17,18] and implemented in the Sage software [19], one can represent a subgroup *G* of Γ either through the permutation representation *P* of the cosets of *G* in Γ (by making use of the Coxeter–Todd algorithm) or through the modular representation of *P*. The permutation representation of a finite index subgroup of the two-generator free group is known as a dessin d’enfant [9,10,11]. The modular picture is possible here since *G* is a subgroup of Γ. Doing this, one arrives at the unexplored relationship of permutation gates of quantum computing, informationally-complete POVMs [10,11] and the aforementioned modular objects.

### 2.2. Minimal Informationally-Complete POVMs and the Pauli Group

In QBism , all the personalist Bayesian properties of probability theory carry over to quantum states; that is, quantum states, like probabilities, are valuations of belief for future experiences [20].

The paper is a continuation of [11] while restricting to permutation gates stemming from subgroups of Γ. Our interest is still the search for minimal informationally-complete (IC)-POVMs derived from appropriate fiducial states under the action of the (generalized) Pauli group.

A POVM is a collection of positive semi-definite operators $\left\{E_{1},\ldots ,E_{m}\right\}$ that sum to the identity. In the measurement of a state ρ, the *i*-th outcome is obtained with a probability given by the Born rule $p\left(i\right)=\mathrm{tr}\left(\rho E_{i}\right)$. For a minimal IC-POVM, one needs $d^{2}$ one-dimensional projectors $\Pi_{i}=|\psi_{i}\rangle \langle \psi_{i}|$, with $\Pi_{i}=dE_{i}$, such that the rank of the Gram matrix with elements $\mathrm{tr}(\Pi_{i}\Pi_{j})$ is precisely $d^{2}$.

A SIC-POVM (the S means symmetric) obeys the remarkable relation [21]:

(1) $${\left|\langle \psi_{i}|\psi_{j}\rangle \right|}^{2}=\mathrm{tr}\left(\Pi_{i}\Pi_{j}\right)=\frac{d\delta_{ij}+1}{d+1},$$

that allows the explicit recovery of the density matrix as in [22] (Equation (29)). The fiducial states for SIC-POVMs are quite complicated to derive (e.g., [23] for an update), and one must work very hard to find anything simple about them [24].

In this paper, we discover minimal IC-POVMs (i.e., whose rank of the Gram matrix is $d^{2}$) and with Hermitian angles ${\left|\langle \psi_{i}|\psi_{j}\rangle \right|}_{i\neq j}\in A=\left\{a_{1},\ldots ,a_{l}\right\}$, a discrete set of values of small cardinality *l*. A SIC is equiangular with |A|=1 and $a_{1}=\frac{1}{\sqrt{d+1}}$. The states encountered below are considered to live in a cyclotomic field $F=Q[\exp\left(\frac{2i\pi }{n}\right)]$, with n=GCD(d,r), the greatest common divisor of *d* and *r*, for some *r*. The Hermitian angle is defined as ${\left|\langle \psi_{i}|\psi_{j}\rangle \right|}_{i\neq j}={∥(\psi_{i},\psi_{j})∥}^{\frac{1}{\deg}}$, where $∥.∥$ means the field norm [25] (pp. 162) of the pair $\left(\psi_{i},\psi_{j}\right)$ in F and deg is the degree of the extension F over the rational field Q [11].

We construct the relevant IC-POVMs using the covariance with respect to the generalized Pauli group. Let *d* be a prime number; the qudit Pauli group is generated by the shift and clock operators as follows:

(2) $$\begin{array}{c} X|j\rangle =|j+1\;\mathrm{mod}\;d\rangle \\ Z|j\rangle =\omega^{j}|j\rangle \end{array}$$

with ω=exp(2iπ/d) a *d*-th root of unity. In dimension d=2, *X* and *Z* are the Pauli spin matrices $\sigma_{x}$ and $\sigma_{z}$. For *N* particles, one takes the Kronecker product of qudit elements *q* times.

Stabilizer states are defined as eigenstates of the Pauli group.

### 2.3. The Single Qubit SIC-POVM

The covariance property under the Pauli group can be illustrated in the two-dimensional case. One can start with the qubit fiducial/magic state $|T\rangle =\cos\left(\beta \right)|0\rangle +\exp\left(\frac{i\pi }{4}\right)\sin\left(\beta \right)|1\rangle$, $\cos\left(2\beta \right)=\frac{1}{\sqrt{3}},$ employed for universal quantum computation [14]. It is defined as the $\omega_{3}=\exp\left(\frac{2i\pi }{3}\right)$—eigenstate of the SH matrix (the product of the Hadamard matrix *H* and the phase gate $S=\left(\begin{array}{cc} 1 & 0\\ 0 & i \end{array}\right)$). Taking the action on $|T\rangle$ of the four Pauli gates *I*, *X*, *Z* and *Y*, the corresponding (pure) projectors $\Pi_{i}=|\psi_{i}\rangle \langle \psi_{i}|,i=1,\ldots ,4$, sum to twice the identity matrix, thus building a POVM, and the pairwise distinct products satisfy $|\langle \psi_{i}|\psi_{j}\rangle {|}^{2}=\frac{1}{3}$. The four elements $\Pi_{i}$ form the well-known two-dimensional SIC-POVM ([21], Section 2).

### 2.4. The Kochen–Specker Theorem

Humans are rats who themselves construct the labyrinth they propose to escape (Rats qui construisent eux-mêmes le labyrinthe dont ils se proposent de sortir.) [26].

In a nutshell, the measured value of a quantum observable sometimes depends on which other mutually-compatible measurements might be performed. This leads to the concept of quantum contextuality. State-independent contextuality is often formulated in terms of the Kochen–Specker (KS) theorem because this theorem is able to guarantee the non-existence of non-contextual hidden variable theories, at least for dimension d≥3. A non-coloring KS proof consists of a finite set of projectors that cannot be assigned truth values (one for true, zero for false) in such a way that (i) one member of each complete orthonormal basis is true and (ii) no two orthogonal (that is, mutually-compatible) projectors are both true; see [27,28,29] and the references therein. Remarkably, it will be shown that subsets of projectors within the IC-POVMs below may sometimes be used to derive proofs of KS theorem (in Dimensions 4, 8 and 9). Contextuality and negativity of the Wigner function happen to play a fundamental role in universal schemes of quantum computation (e.g., [10,20,30,31]).

## 3. Permutation Gates from Γ, Fiducial States and Informationally-Complete Measurements

In all of the paper, we restrict to IC-POVMs that are built from subgroups of the modular group Γ (in contrast to [11], where more general subgroups of the two-generator free groups were also considered).

Using the function “G.aspermutation.group()" in Sage [19], the permutation representation of the group *G* is converted into that of the relevant subgroup $\Gamma_{s}$ of Γ. If $\Gamma_{s}$ is congruence, it can be identified in the Cummins–Pauli table [18] from its features, e.g., the genus *g*, the level *N*, the number of elliptic points of order two $\nu_{2}$, the number of elliptic points of order three $\nu_{3}$, the number of cusps *c* of widths *W*, as well as the fractions of the Farey symbol.

### 3.1. The Three-Dimensional Hesse SIC

**Figure 1:**
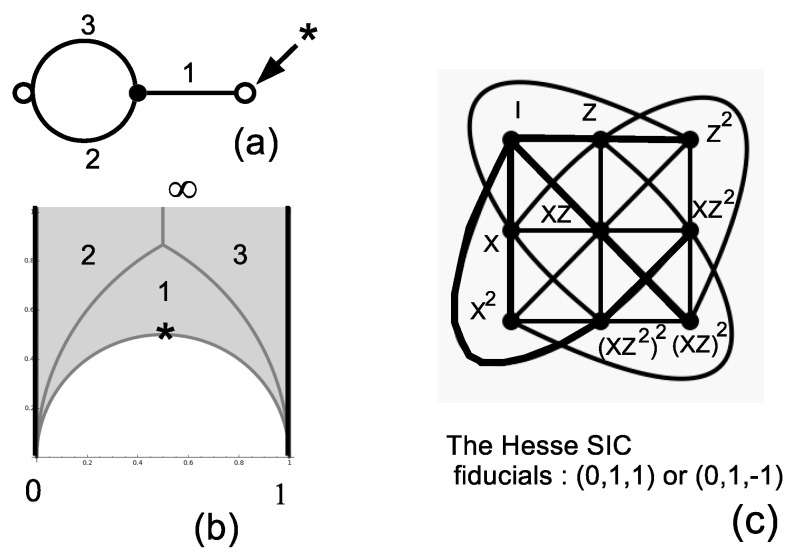
Representation of $S_{3}≅\Gamma_{0}\left(2\right)$ as a dessin d’enfant (**a**) and as the tiling of the fundamental domain (the two thick vertical lines have to be identified) (**b**). The character * denotes the unique elliptic point (of order two). The resulting Hesse SIC-POVM (symmetric informationally-complete positive operator valued measure) is in (**c**).

The only permutation group that can be used to build a three-dimensional IC-POVM (here, an SIC) is the symmetric group $S_{3}=\langle e,v\rangle$ with generators $e=\left(2,3\right)\equiv \left(\begin{array}{ccc} 1 & 0 & 0\\ 0 & 0 & 1\\ 0 & 1 & 0 \end{array}\right)$ and $v=\left(1,2,3\right)\equiv \left(\begin{array}{ccc} 0 & 1 & 0\\ 0 & 0 & 1\\ 1 & 0 & 0 \end{array}\right)$, made explicit in terms of the (Index 3) permutation representation and the corresponding permutation gate. Using Sage and the table of congruence subgroups [18], it is straightforward to recognize that $\Gamma_{s}=\Gamma_{0}\left(2\right)$, whose fundamental domain is pictured in Figure 1b. In this particular case, $\nu_{2}=1$ (the elliptic point at $z=\frac{1}{2}\left(1+i\right)$ is denoted by the symbol *), $\nu_{3}=0$, the cusps are at zero and ∞ and the fractions are at zero and one. The subgroup $\Gamma_{s}=\Gamma_{0}\left(2\right)$ is generated by two transformations $S_{\Gamma_{s}}:z\to \frac{z-1}{2z-1}$ and $T_{\Gamma_{s}}:z\to z+1$.

The eigenstates of the permutation matrices in $S_{3}$ can serve as fiducial states for an IC-POVM as qutrits in the classes $\left(0,1,\pm 1\right)\equiv \frac{1}{\sqrt{2}}(|1\rangle \pm |2\rangle )$. Taking the action of the the nine qutrit Pauli matrices, one arrives at the well-known Hesse SIC. The Hesse SIC is illustrated in Figure 1c: the lines of the configuration correspond to projectors whose traces of triple products equal $\pm \frac{1}{8}$ [32], ([11], Figure 1a). Instead of labeling coordinates as projectors, one labels them with the qutrit operators acting on the fiducial state.

### 3.2. The Two-Qubit IC-POVM

**Figure 2:**
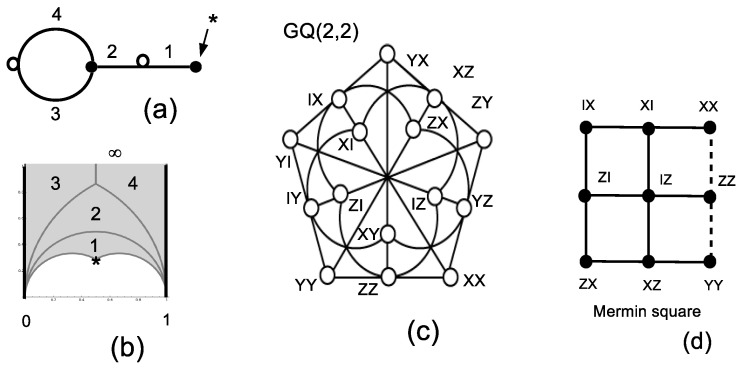
Representation of $A_{4}≅\Gamma_{0}\left(3\right)$ as a dessin d’enfant (**a**) and as the tiling of the fundamental domain (the two thick vertical lines have to identified) (**b**). The character * denotes the unique elliptic point (of order three). The organization of triple products of projectors leads to the generalized quadrangle GQ(2,2) pictured in (**c**) whose subset is the Mermin square (**d**). Traces of triple products for rows (respectively columns) of the Mermin square equal $-\frac{1}{27}$ (respectively $\frac{1}{27}$).

The smaller permutation group that can be used to build a four-dimensional IC-POVM is the alternating group $A_{4}=\langle e,v\rangle$ with generators $e=\left(1,2\right)\left(3,4\right)\equiv \left(\begin{array}{cccc} 0 & 1 & 0 & 0\\ 1 & 0 & 0 & 0\\ 0 & 0 & 0 & 1\\ 0 & 0 & 1 & 0 \end{array}\right)$ and $v=\left(2,3,4\right)\equiv \left(\begin{array}{cccc} 1 & 0 & 0 & 0\\ 0 & 0 & 1 & 0\\ 0 & 0 & 0 & 1\\ 0 & 1 & 0 & 0 \end{array}\right)$, made explicit in terms of the (Index 4) permutation representation and the corresponding permutation gate. Using Sage and the table of congruence subgroups [18] one recognizes that $\Gamma_{s}=\Gamma_{0}\left(3\right)$, whose fundamental domain is pictured in Figure 2b. In this particular case, $\nu_{2}=0$, $\nu_{3}=1$ (the elliptic point at $\frac{1}{2}\left(1+\frac{i}{\sqrt{3}}\right)$ is denoted by the symbol *), the cusps are at zero and ∞ and the fractions are at zero and one. The subgroup $\Gamma_{s}=\Gamma_{0}\left(3\right)$ is generated by two transformations $S_{\Gamma_{s}}:z\to \frac{z-1}{3z-2}$ and $T_{\Gamma_{s}}:z\to z+1$.

The joined eigenstates of the commuting permutation matrices in $S_{3}$ that can serve as fiducial states for an IC-POVM are of the form $\left(0,1,-\omega_{6},\omega_{6}-1\right)\equiv \frac{1}{\sqrt{3}}\left(|01\rangle -\omega_{6}|10\rangle +\left(\omega_{6}-1\right)|11\rangle \right)$, with $\omega_{6}=\exp\left(\frac{2i\pi }{6}\right)$. Taking the action of the two-qubit Pauli group on the latter type of state, the corresponding pure projectors sum to four-times the identity (to form a POVM) and are independent, with the pairwise distinct products satisfying the dichotomic relation $\mathrm{tr}{\left(\Pi_{i}\Pi_{j}\right)}_{i\neq j}=|\langle \psi_{i}|\psi_{j}\rangle {|}_{i\neq j}^{2}\in \left\{\frac{1}{3},\frac{1}{3^{2}}\right\}$. Thus, the 16 projectors $\Pi_{i}$ build an asymmetric informationally-complete POVM (see also [11], Section 2).

The organization of triple products of projectors whose trace is constant (that is, equal to $\frac{1}{9}$ or $\pm \frac{1}{27}$) is that of the generalized quadrangle of order two GQ(2,2) (see [33] and the references therein for more on this concept), as shown in Figure 2c. Simultaneously, the two-qubit operators labeling the vertices of GQ(2,2) are such that the product of operators on a line of GQ(2,2) equals the identity matrix or its negative. By restricting to triples of projectors whose trace is $\pm \frac{1}{27}$, one identifies the standard Mermin square in Figure 2d that is known to allow an operator proof of the Kochen–Specker theorem (e.g., [28]).

Finally, let us observe that the group $S_{4}=\langle (1,2),(2,3,4)\rangle$ may also be used to built the two-qubit IC-POVM as above. It corresponds to the congruence subgroup $4A^{0}$ in the Cummins–Pauli table: it is of Level 4, with $\nu_{2}=2$, $\nu_{3}=1$ and a single cusp at ∞.

### 3.3. The Five-Dimensional Equiangular IC-POVM

**Figure 3:**
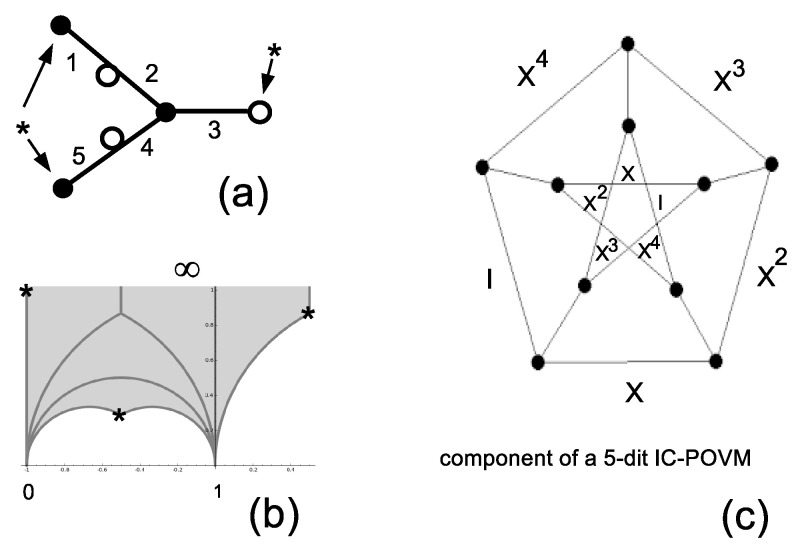
Representation of $A_{5}≅5A^{0}$ as a dessin d’enfant (**a**) and as the tiling of the fundamental domain (**b**). The character * denotes the two elliptic points of order three. (**c**) A one-point intersection graph organizing the lines of the five-dit equiangular informationally-complete positive operator valued measure (IC-POVM) defined from the triple products of constant trace $-\frac{1}{4^{3}}$.

There is just one subgroup of Index 5 (up to conjugation) of the two-generator free group isomorphic to Γ. The organization of cosets defines the alternating group $A_{5}=\langle e,v\rangle$ with generators e=(1,2)(4,5) and v=(2,3,4). Using Sage and the table of congruence subgroups [18], one recognizes that $\Gamma_{s}$ is the congruence subgroup $5A^{0}$ of Level 5 whose fundamental domain is pictured in Figure 3b.

There is one single elliptic point of order two, two elliptic points of order three denoted by the symbol *, one cusp at ∞ and two fractions at −1 and zero. The subgroup $\Gamma_{s}=5A^{0}$ is generated by three transformations $z\to -\frac{z+2}{z+1}$, $z\to -\frac{2z+1}{3z+1}$ and $z\to \frac{1}{1-z}$. Fixed points of such transformations correspond to elliptic points of order two at z=−1+i and order three at $z=\frac{1+i\sqrt{3}}{2}$ and $z=-\frac{1}{2}+\frac{i}{2\sqrt{3}}$.

The joined eigenstates of the commuting permutation matrices in $A_{5}$ that can serve as fiducial states for an IC-POVM are of type (0,1,1,1,1) and (0,1,−1,−1,1). The latter type allows one to construct IC-POVM’s such that the pairwise distinct products satisfy $|\langle \psi_{i}|\psi_{j}\rangle {|}^{2}=\frac{1}{4^{2}}$, that is the POVM is equiangular with respect to the field norm defined in the Introduction. The first type of magic state is dichotomic with values of the products $\frac{1}{4^{2}}$ and ${\left(\frac{3}{4}\right)}^{2}$. The trace of pairwise products of (distinct) projectors is not constant. For example, with the state (0,1,−1,−1,1), one gets a field norm equiangular IC-POVM in which the trace is trivalued: it is either 1/16 or $(7\pm 3\sqrt{5})/32$.

Let us concentrate on the equiangular POVM. Traces of triple products with constant value $-\frac{1}{4^{3}}$ define lines organized into a geometric configuration of type $\left(25_{12},100_{3}\right)$. Lines of the configuration have one or two points in common. The two-point intersection graph consists of 10 disjoint copies of the Petersen graph. One such Petersen graph is shown in Figure 3c; the vertices of the graph correspond to the lines, and the edges correspond to the one-point intersection of two lines. As before, the labeling is in terms of the operators acting on the magic state.

### 3.4. The Six-Dimensional IC-POVM

One finds five distinct permutation groups of Index 6 corresponding to subgroups of Γ that lead to a six-dimensional IC-POVM. Fiducial states are of type $\left(0,1,\omega_{6}-1,0,-\omega_{6},0\right)$ already found in [11] with $\mathrm{tr}{\left(\Pi_{i}\Pi_{j}\right)}_{i\neq j}=|\langle \psi_{i}|\psi_{j}\rangle {|}_{i\neq j}^{2}=\frac{1}{3}\;\mathrm{or}\;\frac{1}{3^{2}}$.

**Figure 4:**
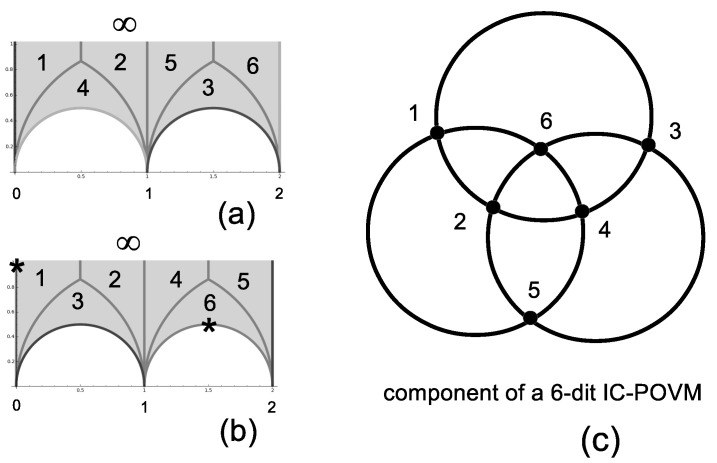
(**a**) Fundamental domain for the genus 1 group $\Gamma^{\prime }$; (**b**) fundamental domain for the genus 0 group $6A^{1}$; the symbol * points out the two elliptic points of order two; (**c**) a basic piece of the six-dit IC-POVM with fiducial state of type $\left(0,1,\omega_{6}-1,0,-\omega_{6},0\right)$ obtained through the action of Pauli operators 1–6: the lines correspond to four-tuple products of projectors with constant trace $\frac{1}{9}$ and simultaneously of products equal to ±I. There are two disjoint copies looking like Borromean rings with points as $\left[1\ldots 6\right]=\left[I,ZX^{3},Z^{2},Z^{3}X^{3},Z^{4},Z^{5}X^{3}\right]$ (for lines with projector products −I) and $\left[1\ldots 6\right]=\left[X^{4},Z,Z^{2}X^{3},Z^{3},Z^{4}X^{3},Z^{5}\right]$ (for lines with projector products *I*).

The five permutation groups under question are the cyclic group $Z_{6}$ leading to congruence subgroups $\Gamma^{\prime }$ and Γ(2), the alternating group $A_{4}$ leading to the congruence subgroup $3C^{0}$ [18] and the symmetric group $S_{4}$ leading to the congruence subgroups $\Gamma_{0}\left(4\right)$ and $\Gamma_{0}\left(5\right)$. Fundamental domains for $\Gamma^{\prime }$ and $3C^{0}$ are shown in Figure 4a,b, respectively.

For a six-dimensional IC-POVM, one discovers a quite simple geometry sustaining the four-tuple products of projectors having constant trace $\frac{1}{9}$ and simultaneously having products of corresponding operators on a line equal to ±I. It consists of two disjoint copies (corresponding to lines whose product of projectors equal −I and *I*, respectively) looking like Borromean rings, as shown in Figure 4.

### 3.5. Seven-Dimensional IC-POVMs

Seven-dimensional IC-POVMs with bivalued pairwise products $|\langle \psi_{i}|\psi_{j}\rangle {|}_{i\neq j}^{2}$ are found starting from permutation groups isomorphic to $Z_{7}⋊Z_{6}$ or PSL(2,7), respectively. The first permutation group corresponds to a non-congruence subgroup. The fiducials of the IC are of type (0,1,1,1,±1,±1,±1) or $\left(0,1,-\omega_{3}-1,\omega_{3},1,-\omega_{3}-1,\omega_{3}\right)$. The second permutation group corresponds to the congruence subgroup $7A^{0}$ [18]. The fiducials of the IC are of type (1,0,0,0,1,±1,±1) or (1,0,0,0,i,i,1).

**Figure 5:**
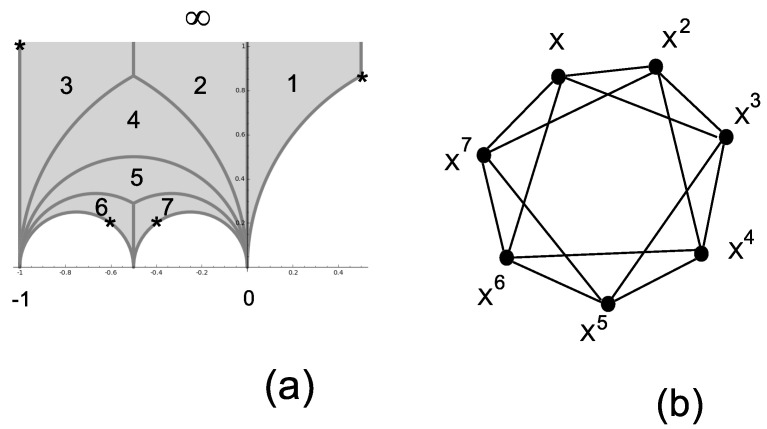
(**a**) Fundamental domain for the group $7A^{0}$; (**b**) a basic component associated with a bivalued seven-dimensional IC-POVM.

The fundamental domain for the group $7A^{0}$ is shown in Figure 5a. The geometry of IC triple products is quite complex, but one building block may be identified as shown in Figure 5b (only for ICs with non-complex entries in their fiducial).

It may be reminded that an equiangular (with respect to the cyclotomic field norm) seven-dimensional IC-POVM exists. It is obtained thanks to the group $Z_{7}⋊Z_{6}$ (in a non-modular representation) using magic permutations [11]. A fiducial such as $\left(1,-\omega_{3}-1,-\omega_{3},\omega_{3},\omega_{3}+1,-1,0\right)$ does the job with $|\langle \psi_{i}|\psi_{j}\rangle {|}_{i\neq j}^{2}=\frac{1}{6^{2}}$.

### 3.6. The Three-Qubit Hoggar SIC

The approach based on permutation groups fails to identify any IC-POVM; see [16] for details about the geometry of the Hoggar SIC in relation to its covariance under the three-qubit Pauli group. A noticeable result is that the three-qubit SIC embeds the dual of the generalized hexagon GH(2,2) [11,16] (Figure 3). The latter geometry is connected to the eight-dimensional Kochen–Specker theorem.

### 3.7. Nine-Dimensional IC-POVMs

**Figure 6:**
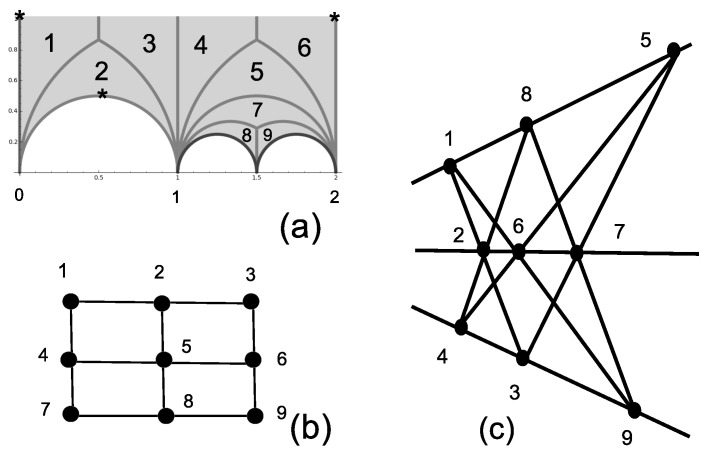
(**a**) Fundamental domain for the non congruence subgroup of Γ associated with the group $Z_{3}^{2}⋊\left(Z_{2}⋊S_{4}\right)$. (**b**) A basic component (a (3×3)-grid) in the geometry of the bivalued nine-dimensional IC-POVM with fiducial (1,0,0,0,1,0,1,1,0). Vertices of the grid for observables are the two-qutrit observables $\left[1,\cdots ,9\right]=\left[I⊗X,I⊗XZ,I⊗XZ^{2},Z⊗X,Z⊗XZ,Z⊗XZ^{2},Z^{2}⊗X,Z^{2}⊗XZ,Z^{2}⊗XZ^{2}\right]$. (**c**) A basic component (a Pappus configuration) in the geometry of the bivalued nine-dimensional IC-POVM with fiducial (1,0,0,0,−1,0,−1,1,0). The points are $\left[1,\cdots ,9\right]=[I⊗Z,I⊗XZ,I⊗{\left(XZ^{2}\right)}^{2},$$Z⊗I,Z⊗X,Z⊗X^{2},Z^{2}⊗Z^{2},Z^{2}⊗{\left(XZ\right)}^{2},Z^{2}⊗XZ^{2}]$.

The smallest permutation group isomorphic to a subgroup of Γ and useful to build a nine-dimensional IC-POVM is $P=\langle (1,2,3)(4,5,6)(7,8,9),(3,4)(5,7)(8,9)\rangle$$≅Z_{3}^{2}⋊\left(Z_{2}⋊S_{4}\right)$. The corresponding fundamental domain is shown in Figure 6a, the subgroup is non-congruence and contains three elliptic points of order two at *i*, 2+i and (1+i)/2 and two cusps at $\frac{3}{2}$ and ∞. The related IC-POVM has bi- or three-valued distinct pairwise products. Let us choose the fiducial state for the bivalued case as (1,0,0,0,±1,0,0,±1,1,0). For the state with positive entries and traces of triple products equal to $-\frac{1}{8}$, the geometry consists of six copies of a 3×3 grid, as shown on Figure 6b. The products of the observables on a row or a column of the grid equal one, $\omega_{3}$ or $\omega_{3}^{2}$. For the state with positive and negative entries and traces of triple products equal to $\frac{1}{8}$, the geometry consists of nine copies of the Pappus configuration, as shown in Figure 6c. It allows the proof of the 2-qutrit Kochen–Specker theorem [11] (Section 2.7 and Figure 5).

A trivalued IC-POVM follows from the permutation group of order 504 that is also non-congruence. The fiducial is of type (0,1,1,1,1,1,1,1,1).

It is useful to remind that a group generated by two magic permutations and isomorphic to $Z_{3}^{2}⋊Z_{4}$ has been used to construct a bivalued IC-POVM starting from a fiducial of type (1,1,0,0,0,0,−1,0,−1) [11]. Noticeably, It contains the Pappus configuration in the organization of its triple products.

### 3.8. Higher Dimensional IC-POVMs

IC-POVMs found in dimension *d* higher than nine are summarized in the second half of Table 1. The minimal number of pairwise products needed increases with *d* (as in Column 3), and the subgroup $\Gamma_{s}$ occurring in the construction is quite often non-congruence (as expressed in Column 2). The symmetry underlying triple products of projectors is not simple and not easily recognizable. We could not find (modular group based) IC-POVMs in dimensions d=8,16,17,22,23. In Dimension 24, the found ICs are covariant under the 24-dit Pauli group, not under the 3QB-QT group. Finally, in Dimension 27, one finds a four-valued IC-POVM, covariant under the 3QT Pauli group, whose structure of the triple products consists of 81 copies of the Pappus configuration.

Table 1 summarizes the results obtained so far.

## 4. Conclusions

It would be nice if we could design a virtual reality in Hyperbolic Space, and meet each other there [34].

The continuing search of mathematical structures governing the weirdness of quantum theory is catalyzed by applications. A universal quantum computer needs non-stabilizer states, i.e., states that are not eigenstates of a Pauli group. The finding of distillable qubit magic states in [14] prompted us to pass to higher dimensions with the methods of permutation theory [10]. It was soon observed that an interesting subset of magic states could be seen as fiducials for minimal IC-POVMs of the corresponding dimension [11]. Now, the present work connects universal quantum computing, IC-POVMs, quantum contextuality and the subgroups of finite index $\Gamma_{s}$ of the modular group Γ. This allows one to see the magic/fiducial states arising from appropriate permutation gates in the new language of fundamental domains and their copies under the discontinuous action of $\Gamma_{s}$. Thus and unexpectedly, a short circuit occurs between Γ and quantum theory that has not been investigated so far. The group Γ is the starting point of the modularity theorem that connects elliptic curves over the rationals and modular forms. A jewel of mathematics is tethered to our best physical theory.

## Figures and Tables

**Table 1:** A summary of the subgroups of the modular group Γ (Column 2) allowing the construction of informationally-complete positive operator valued measures (IC-POVMs) in the corresponding dimension (Column 1). When non-congruence (denoted NC), the signature NC $\left(g,N,\nu_{2},\nu_{3},\left[c_{i}^{W_{i}}\right]\right)$ is made explicit. Column 3 shows the minimal number of pairwise distinct products needed (denoted PP).

Dim	Subgroups of Γ Leading to an IC-POVM	PP	Geometry
2	none	1	tetrahedron [14]
3	$\Gamma_{0}\left(2\right)$	1	Hesse SIC [15]
4	under 2QB Pauli group		
	$\Gamma_{0}\left(3\right),4A^{0}$	2	GQ(2,2)
5	$5A^{0}$	1	Petersen graph
6	$\Gamma^{\prime },\Gamma \left(2\right),3C^{0},\Gamma_{0}\left(4\right),\Gamma_{0}\left(5\right)$	2	Borromean ring
7	$7A^{0}$	2	Figure 5b
	NC$\left(0,6,1,1,\left[1^{1}6^{1}\right]\right)$	2	
	none	1	[11]
8	none under 3QB, 8-dit, 4-dit-QB Pauli group	1	Hoggar SIC [11,16]
9	under 2QT Pauli group		
	NC$\left(0,8,3,0,\left[1^{1}8^{1}\right]\right)$	2	(3×3)-grid, Pappus
	NC$\left(0,9,1,3,\left[9^{1}\right]\right)$	3	$\left[81_{8},216_{3}\right]$
10	$5C^{0}$	5	
11	$11A^{0}$	3	$\left[11_{3}\right]$
12	under 2QB-QT Pauli group		
	$10A^{1}$	5	K(3,3,3,3)
	NC $\left(0,8,4,0,\left[4^{1}8^{1}\right]\right)$	5	Hesse (×16)
	NC$\left(0,8,4,0,\left[4^{1}8^{1}\right]\right)$	6	$\left[48_{7},112_{3}\right]$
12	under 12-dit Pauli group		
	$8A^{1}$, NC$\left(0,8,4,0,\left[4^{1}8^{1}\right]\right)$	11,7	
13	NC$\left(0,6,1,1,\left[1^{1}6^{2}\right]\right)$	4	
14	$7C^{0}$, NC$\left(0,6,0,2,\left[1^{1}6^{2}\right]\right)$, $14A^{1}$	12,5,6	
15	$5E^{0}$, NC$\left(0,6,3,0,\left[3^{1}6^{2}\right]\right)$, $15A^{1}$, $10B^{1}$	5,4,10,3	
16	none under 4QB and 2 4-dit Pauli group		
18	under 18-dit or 2QT-QB Pauli group		
	$\Gamma_{0}\left(10\right)$, NC$\left(1,8,0,0,\left[2^{1}8^{2}\right]\right)$	7,5	
19	NC$\left(0,6,1,1,\left[1^{1}6^{3}\right]\right)$	3	
21	NC$\left(0,6,3,0,\left[3^{1}6^{3}\right]\right)$, NC$\left(0,6,1,0,\left[1^{1}2^{1}6^{3}\right]\right)$	4	
	NC$\left(0,14,7,0,\left[7^{1}14^{1}\right]\right)$, NC$\left(0,8,3,0,\left[1^{1}4^{1}8^{2}\right]\right)$	59,4	
24	none under 3QB-QT Pauli group		
24	under 24-dit Pauli group		
	$24A^{1}$, NC$\left(2,12,0,0,\left[12^{2}\right]\right)$, $20B^{1}$, $12F^{1}$	40,56,40,30	
	NC$\left(0,6,2,0,\left[3^{2}6^{3}\right]\right)$, NC$\left(1,8,0,0,\left[4^{2}8^{2}\right]\right)$	8,7	
	$21A^{2}$, $24A^{1}$	23,60	
25	under 25-dit Pauli group		
	NC$\left(0,10,5,1,\left[5^{1}10^{2}\right]\right)$	15	
27	under 3QT Pauli group		
	NC$\left(0,6,1,0,\left[1^{1}2^{1}6^{4}\right]\right)$, NC$\left(0,8,3,0,\left[1^{1}2^{1}8^{3}\right]\right)$	4	Pappus
